# Validating biologic age in selecting elderly patients with pancreatic cancer for surgical resection

**DOI:** 10.1002/jso.27121

**Published:** 2022-11-02

**Authors:** Anthony J. Scholer, Rebecca Marcus, Mary Garland‐Kledzik, Shu‐Chin Chang, Adam Khader, Juan Santamaria‐Barria, Zeljka Jutric, Ronald Wolf, Melanie Goldfarb

**Affiliations:** ^1^ Division of Surgical Oncology University of South Carolina School of Medicine Greenville South Carolina USA; ^2^ Department of Surgery Saint John's Cancer Institute at Providence St. John's Health Center Santa Monica California USA; ^3^ Division of Surgical Oncology West Virginia University Morgantown West Virginia USA; ^4^ Department of Surgery Medical Data Research Center, Providence Saint Joseph Health Oregon Portland USA; ^5^ Department of Surgery, Division of Surgical Oncology Hunter Holmes McGuire Veterans Affair Medical Center Richmond Virginia USA; ^6^ Department of Surgery, Division of Surgical Oncology University of Nebraska Medical Center Omaha Nebraska USA; ^7^ Department of Surgery, Division of Hepatobiliary and Pancreas Surgery and Islet Cell Transplantation University of California Irvine Medical Center Orange California USA

**Keywords:** deficit accumulation frailty index, distal pancreatectomy, pancreas resection, whipple

## Abstract

**Background and Objectives:**

Selecting frail elderly patients with pancreatic cancer (PC) for pancreas resection using biologic age has not been elucidated. This study determined the feasibility of the deficit accumulation frailty index (DAFI) in identifying such patients and its association with surgical outcomes.

**Methods:**

The DAFI, which assesses frailty based on biologic age, was used to identify frail patients using clinical and health‐related quality‐of‐life data. The characteristics of frail and nonfrail patients were compared.

**Results:**

Of 242 patients (median age, 75.5 years), 61.2% were frail and 32.6% had undergone pancreas resection (surgery group). Median overall survival (mOS) decreased in frail patients (7.13 months, 95% confidence interval [CI]: 5.65–10.1) compared with nonfrail patients (16.1 months, 95% CI: 11.47–34.40, *p* = 0.001). In the surgery group, mOS improved in the nonfrail patients (49.4%; 49.2 months, 95% CI: 29.3–79.9) compared with frail patients (50.6%, 22.1 months, 95% CI: 18.3–52.4, *p* = 0.10). In the no‐surgery group, mOS was better in nonfrail patients (54%; 10.81 months, CI 7.85–16.03) compared with frail patients (66%; 5.45 months, 95% CI: 4.34–7.03, *p* = 0.02).

**Conclusions:**

The DAFI identified elderly patients with PC at risk of poor outcomes and can identify patients who can tolerate more aggressive treatments.

**Abbreviations**
aHR: adjusted hazard ratio
DAFI: deficit‐accumulation‐frailty‐index
HRQOL: health‐related quality of life
MAO: Medicare Advantage Organization
mDAFI: median deficit‐accumulation‐frailty‐index
MHOS: The Medicare Health Outcomes Survey
mOS: median overall survival
OS: overall survival
PC: pancreas cancer
pDAFI: predictive deficit‐accumulation‐frailty‐index
*r*
: correlation coefficient
*r* ^2^
: coefficient of determination
SEER: surveillance, epidemiology and end results

## 1 INTRODUCTION

The difficulty in determining optimal treatment pathways for elderly patients with pancreatic cancer (PC) is that during PC treatment, the body endures stress resulting in limited physiologic reserve independent of chronologic age, and organ dysfunction may develop subsequently.^1^, ^2^, ^3^, ^4^, ^5^ Therefore, the biologic age of patients with PC should be determined to understand baseline physiologic reserve and appropriately tailor individual treatment recommendations rather than using the traditional measurements of frailty that are dependent on chronologic age.^6^, ^7^, ^8^, ^9^

With the increasing “baby boomers” population and improvements in healthcare, the United States is moving toward an elderly population. In 2016, 46 million people were aged 65 years or older, and an estimated increase to 64 million (1 in 5 Americans) by 2030 and 90 million by 2050 is anticipated.^10^, ^11^ The incidence of cancer increases with age. For PC, specifically, the majority of patients (89.1%) are ≥65 years old at the time of diagnosis (diagnosed at ages 55–64 years, 22.0%; at ages 65–74 years, 29.2%; at ages 75–84 years, 24.4%; and at >84 years, 13.5%).^12^ In 2021, there were approximately 60 430 new PC diagnoses, making it the 10th most common cancer diagnosis in males (31 950 diagnoses, 3% of all cancers), the eighth most common cancer diagnosis in females (28 480 diagnoses, 3% of all cancers), and the fourth most common cause of cancer‐related mortality in the United States (48 220 total PC deaths).^13^, ^14^

The optimal treatment regimen for older patients, defined in this study as patients aged >65 years old, with PC is currently unknown. The challenge with treating these older patients is the ability to accurately identify those who can tolerate more aggressive treatment regimens (pancreatic resection with or without chemotherapy and/or radiation) while simultaneously identifying patients who may develop significant morbidity and a decline in health‐related quality of life (HRQOL) with such aggressive treatment regimens.^4^, ^5^, ^15^, ^16^, ^17^, ^18^, ^19^, ^20^, ^21^

Rockwood et al. proposed a concept of biologic age, known as the deficit‐accumulation frailty index (DAFI), where an individual's health status can be quantified as a proportion of aging‐associated deficits the individual has developed.^2^, ^22^, ^23^, ^24^ The DAFI, compared with other measures of frailty, may better characterize a decline in function among elderly patients without cancer.^9^, ^25^, ^26^, ^27^, ^28^ Furthermore, the DAFI has been found to predict treatment‐related toxicity, drug discontinuation, patient hospitalization, and mortality in certain cancers; thus, it has the potential to be used as a clinical screening tool in elderly patients with PC.^29^, ^30^, ^31^, ^32^, ^33^

This study aimed to determine the feasibility of constructing a pancreas cancer‐specific DAFI, investigate the DAFI's association with pancreas resection outcomes, and develop a screening tool for the appropriate selection of elderly patients with PC for more aggressive oncologic treatment regimens.

## 2 MATERIALS AND METHODS

### 2.1 Data source

The surveillance, epidemiology, and end results (SEER) cancer registry contains clinical, demographic, and cause of death information for oncologic patients from geographical regions.^34^, ^35^ The Medicare Health Outcomes Survey (MHOS) contains self‐reported HRQOL data for patients enrolled in the Medicare Advantage Organization (MAO). The linked SEER‐MHOS database uses identifiers from MHOS surveys that are matched to identifiers within the SEER‐Medicare database. At the time of writing this article, the SEER‐MHOS database consisted of 18 cohorts of MHOS data including patient baseline and follow‐up information from 1998 to 2017, SEER cancer diagnoses from 1998 to 2015, and SEER vital statuses from 1998 to 2013.^34^, ^35^, ^36^ This linked database only includes information from SEER patients who have completed at least one MHOS survey. It does not include patients who are Medicare beneficiaries in a fee‐for‐service program or Medicare managed care beneficiaries who are not included in a MHOS cohort. Additionally, the database does not include information on chemotherapy or hormonal treatment regimens, cancer recurrence, Medicare claims, or prescription drug information.

### 2.2 Patient cohorts

**Figure 1:**
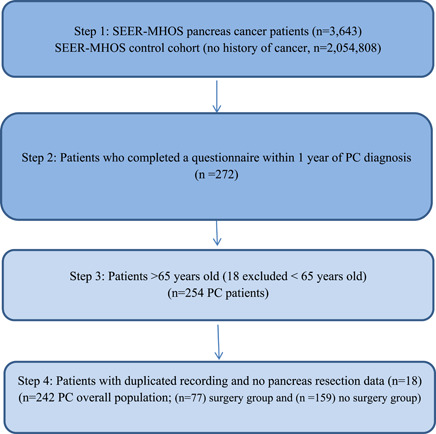
CONSORT diagram for the pancreatic cancer cohort. PC, pancreatic cancer; SEER‐MHOS, surveillance, epidemiology, and end results‐Medicare Health Outcomes Survey. [Correction added on 18 January 2023, Figure 1 has been modified].

Two cohorts of patients with ages >65 years were identified from the 1998 to 2017 SEER‐MHOS database (Figure 1). The first, representing the control group (no history of cancer), consisted of patients without oncologic diagnoses. The second patient cohort included patients who were older than 65 years with all stages of PC (overall PC population). Patients were included in this cohort only if they had completed a MHOS survey within 1 year after their PC diagnoses, which served as an estimate of baseline frailty. To control for potential confounders before diagnosis, patients were excluded if they had filled out a survey before their diagnosis of PC. This study was approved by the Institutional Review Board of Saint John's Cancer Institute at Providence St. John's Health Center, before the data being released to the authors' institution.

### 2.3 Development of a DAFI for the control group

We followed the standard procedure described by Mitnitski et al.^2^ to construct the DAFI. A 25‐variable score was constructed for each patient within the control group (noncancer patients) using previously described and validated methodology.^2^, ^31^, ^37^ The variables used to calculate these scores were biologically relevant and within the following five categories: activities of daily living, chronic health conditions, functionality, general health, and mental health. Each variable was quantified using a numeric score: 1 (present), 0.5 (limited), or 0 (absent)^32^, ^38^ (Table 1).

**Table 1:** Variables included in the pancreas cancer deficit accumulation frailty index

Variables	Index Score		
	0	0.5	1
Activities of daily living			
Difficulty bathing	No, I do not have difficulty	Yes, I have difficulty	I am unable to do this activity
Difficulty dressing	No, I do not have difficulty	Yes, I have difficulty	I am unable to do this activity
Difficulty eating	No, I do not have difficulty	Yes, I have difficulty	I am unable to do this activity
Difficulty using the toilet	No, I do not have difficulty	Yes, I have difficulty	I am unable to do this activity
Chronic health conditions			
Angina pectoris/coronary artery disease	No		Yes
Hypertension	No		Yes
Myocardial infarction	No		Yes
Congestive heart failure	No		Yes
Stroke	No		Yes
Emphysema, asthma, or chronic obstructive pulmonary disease	No		Yes
Diabetes, high blood sugar, or sugar in urine	No		Yes
Crohn's disease, ulcerative colitis, or inflammatory bowel disease	No		Yes
Arthritis of the hip/knee	No		Yes
Functioning			
Incontinence	No		Yes
Limited in moderate activities	No, not limited at all	Yes, limited a little	Yes, limited a lot
Hearing (hear most things people say)	All of the time	Some of the time	None of the time
Vision (see well enough to read a newspaper)	All of the time	Some of the time	None of the time
Difficulty getting in/out of chairs	No, I do not have difficulty	Yes, I have difficulty	I am unable to do this activity
Difficulty walking	No, I do not have difficulty	Yes, I have difficulty	I am unable to do this activity
Difficulty climbing several flights of stairs	No, I do not have difficulty	Yes, I have difficulty	I am unable to do this activity
General health			
Lots of energy	All of the time	Some of the time	None of the time
General health	Excellent	Good	Poor
Pain interfering with work	Not at all	Some of the time	Quite a bit
Mental health			
Calm and peaceful	All of the time	Some of the time	None of the time
Downhearted and blue	None of the time	Some of the time	All of the time

To calculate the mean DAFI (mDAFI) for each chronologic age, we first calculated the DAFI for each participant by dividing the sum of a participant's deficit scores by the total potential score of the same participant.^2^, ^31^ That is:

mDAFI = (#ofindividualvariablesobtained)/(total#ofpotentialvariables),

mDAFI = (actualdeficitscore)/(totalpotentialdeficitscore).

The total potential score of a patient is determined based on the number of variables with available data, as many patients did not respond to the MHOS survey questions pertaining to all 25 variables (i.e., for a patient who answered questions pertaining to 20 of the 25 variables, the total potential score is 20). The DAFI has a theoretical maximum of 1.0, with a score of 0.0 corresponding to no deficits being noted. In this study, only patients with at least 10 of 25 completed MHOS survey questions were included to construct the DAFI.

Next, the mDAFI score for each year of chronologic age was calculated by dividing the sum of all DAFI obtained for patients of a particular age by the total number of patients of that age. Predicted DAFI (pDAFI) was obtained by performing linear regression of the mDAFI on age, which enabled assessment of the correlation between DAFI and chronologic age. Finally, the average pDAFI for each year of chronologic age was used to create binary cutoffs that defined frailty for individual patients. That is, a patient was determined to be frail relative to other patients of the same chronologic age based on whether the individual's DAFI exceeded the average pDAFI of the patient cohort.

### 2.4 Application of DAFI in patients with PC

All patients ages >65 years old with newly diagnosed PC and who had completed a MHOS survey within 1 year of their initial diagnosis, were included in our analyses. DAFI was calculated for individuals within this PC cohort, and frailty statuses were assigned using the cutoff values determined in the control cohort (no history of cancer) analyses. Specifically, a patient with PC was defined as “frail” if that patient's DAFI was greater than the average pDAFI of the control cohort of the same chronologic age and “nonfrail” if that patient's DAFI was less than or equal to the average pDAFI of the control cohort.^2^, ^31^

To understand the relationship of DAFI with chronologic age in patients with PC, the mDAFI and frailty prevalence were reported for multiple age cohorts (66–70, 71–75, 76–80, and ≥81 years), as well as by sex. Next, a mDAFI was estimated for each year of chronologic age within the PC cohort, and a linear regression model of mDAFI with chronologic age as an independent variable was fitted to determine the PC cohort pDAFI. The coefficient of determination, which represents the proportion of the variance in the dependent variable (mDAFI) that is predicted from the independent variable (chronological age), was also calculated from this linear regression.

The Kaplan–Meier method and log‐rank test, followed by multivariable Cox proportional‐hazards regression analyses, were used to evaluate the association between frailty and overall survival (OS). We defined OS as the time from survey completion after PC diagnosis to death or last known contact date. Hazard ratios (HRs) and 95% confidence intervals (CIs) were reported for the overall PC population and by age, sex, race, frailty status, and inclusion of pancreas resection (surgery group) in the treatment regimen. Subgroup analyses of the surgery and no surgery groups were performed to evaluate their association with frailty and OS. All analyses were performed using R version 3.6.0 (R core Team 2019).

## 3 RESULTS

### 3.1 Noncancer patient cohort (control cohort)

The MHOS database contained 2 054 808 individuals (no history of cancer) that were included in our control group (Figure 1, Table 2). The median age of this cohort was 73.2 years (interquartile range [IQR]: 69.1–78.9); 59.7% of the patients were female, and 75.9% were white, non‐Hispanic. The mDAFI score for the control cohort was 0.23 (SD 0.17), and the scores displayed a right‐skewed distribution (skewness value, 0.93). The mDAFI for each year of chronologic age was calculated and found to be strongly correlated with chronologic age (correlation coefficient [*r*] = 0.98; coefficient of determination (*r* ^2^) = 0.95; *p* < 0.001). pDAFI scores were obtained by a fitted linear regression of mDAFI on chronologic age and ranged from 0.15 to 0.53. On average, mDAFI increased by 0.01 (0.03 on a log scale) with each 1‐year increase in chronologic age. Furthermore, each 10% increase in DAFI (approximately three or four additional deficits) was associated with a 42% increased risk of death (adjusted hazard ratio [aHR]: 1.42; 95% CI: 1.41–1.42, *p* < 0.001) when controlling for age, sex, and race.

**Table 2:** Comparison of patient characteristics between those with no history of cancer and with pancreas cancer

	Control cohort (*n* = 2 054 808)	Pancreatic cancer cohort (*n* = 242)	*p* Value
Age, year, median (IQR)	73.2 (69.1–78.9)	75.5 (70.0–80.0)	<0.001
Gender, *n* (%)			0.30
Female	1 226 267 (59.7)	136 (56.2)	
Male	828 541 (40.3)	106 (43.8)	
Race, *n* (%)			0.02
Non‐White	496 091 (24.1)	74 (30.6)	
White	1 558 575 (75.9)	168 (69.4)	
Missing	142 (0)	0 (0)	
Education, *n* (%)			0.07
High school or less	1 257 699 (61.2)	132 (54.5)	
College	575 411 (28.0)	75 (31.0)	
More than college	165 638 (8.1)	27 (11.2)	
Missing	*	*	
Housing, *n* (%)			0.39
Self‐owned/family	1 530 906 (74.5)	170 (70.2)	
Not owned/rented	353 469 (17.2)	46 (19.0)	
Missing	170 433 (8.3)	26 (10.7)	
Income, *n* (%)			0.81
<$10 000	267 960 (13.0)	28 (11.6)	
$10 000–$29 999	760 837 (37.0)	90 (37.2)	
$30 000–$49 999	339 789 (16.5)	36 (14.9)	
≥$50 000	251 146 (12.2)	34 (14.0)	
Unknown	435 076 (21.2)	54 (22.3)	
Frailty, *n* (%)			
Yes	NA	148 (61.2%)	
No	NA	94 (38.8%)	
Pancreas Resection, *n* (%)			
Yes (surgery group)	NA	77 (32.6%)	
No (no surgery group)	NA	159 (67.4%)	
Unknown	*	*	
Pancreas resection, *n* (%)			
Frail (surgery group)	NA	39 (50.6%)	
Nonfrail (surgery group)	NA	38 (49.4%)	
No pancreas resection, *n* (%)			
Frail (no surgery group)	NA	105 (66.0%)	
Nonfrail (no surgery group)	NA	54 (34.0%)	

Abbreviations: IQR, interquartile range; NA, not applicable.

* *n* = <10 so data cannot be analyzed per SEER‐MHOS guidelines. [Correction added on 18 January 2023, a footnote was added and data corresponding to it has been updated in Table 2].

### 3.2 PC cohort (overall PC population)

A total of 3643 patients with PC were identified in the SEER‐MHOS database. Of these patients, 242 were >65 years old, had completed a MHOS questionnaire within 1 year of PC diagnosis and had completed questions pertaining to at least 10 of the 25 variables. The median age of the study cohort was 75.5 years (IQR: 70.0–80.0), with most patients being female (56.2%) and White, non‐Hispanic (69.4%) (Table 2). Regarding age, 25.6% of patients with PC were aged 66–70 years, 24.4% aged 71–75 years, 26.4% aged 76–80 years, and 23.6% aged ≥ 81 years. The mDAFI for all patients with PC was 0.30 (SD 0.18), and the scores displayed a right‐skewed distribution (skewness value, 0.58). The mDAFI increased significantly from 0.28 in patients aged ≤70 years to 0.38 in those aged ≥81 years (*p* = 0.01; Table 3). When assessed by sex, the trend in mDAFI remained significant in female patients with PC aged ≥81 years (*p* = 0.01) but not in male patients (*p* = 0.16; Table 4). For each 1‐year increment, the mDAFI of patients with PC increased by 0.01 (0.03 on a log scale). Compared to the control cohort (no history of cancer), the mDAFI in the PC cohort was weakly correlated with chronologic age (*r* = 0.77, *r* ^2^ = 0.57, *p* < 0.001).

**Table 3:** Deficit accumulation frailty index and frailty by age and sex in patients with pancreas cancer

	DAFI score, mean (SD)			Frailty prevalence, *n* (%)		
**Age group (years)**	**Overall**	**Female**	**Male**	**Overall**	**Female**	**Male**
66−70 (*n* = 62)	0.28 (0.19)	0.28 (0.19)	0.28 (0.19)	45 (72.6)	24 (72.7)	21 (72.4)
71−75 (n = 59)	0.27 (0.17)	0.25 (0.18)	0.30 (0.17)	33 (55.9)	14 (43.8)	19 (70.4)
76−80 (*n* = 64)	0.29 (0.17)	0.33 (0.18)	0.25 (0.15)	34 (53.1)	20 (58.8)	14 (46.7)
81−96 (*n* = 57)	0.38 (0.18)	0.39 (0.18)	0.36 (0.18)	36 (63.2)	25 (67.6)	11 (55)
*p* Value	0.01^a^	0.01^a^	0.16^a^	0.12^b^	0.08^b^	0.14^b^

Abbreviations: ANOVA, analysis of variance; DAFI, deficit accumulation frailty index.

^a^ ANOVA analysis

^b^ *χ* ^2^ test, DAFI.

**Table 4:** Predictive factors of overall survival in elderly patients with pancreatic cancer

	Univariate analysis			Multivariate analysis		
	HR	95% CI	*p* Value	HR	95% CI	*p* Value
Age, years	1.03	1.01–1.06	0.002	1.02	0.99–1.04	0.13
Sex						
Female	Reference			Reference		
Male	1.04	0.80–1.35	0.78	0.93	0.69–1.23	0.60
Race						
Non‐White	Reference			Reference		
White	1.21	0.91–1.60	0.19	1.14	0.85–1.52	0.38
Surgery						
No (no surgery group)	Reference			Reference		
Yes (surgery group)	0.44	0.33–0.58	<0.001	0.46	0.34–0.63	<0.001
Unknown	0.83	0.36–1.88	0.65	0.8	0.35–1.82	0.59
Frail patients with pancreatic cancer						
No	Reference			Reference		
Yes	1.56	1.19–2.05	0.001	1.46	1.11–1.92	0.006

Abbreviations: CI, confidence interval; HR, Hazard ratio.

### 3.3 Association of DAFI with OS in patients with PC

Of the 242 patients with PC, 94.6% had died at the time of data cutoff. The median follow‐up time was 10.1 months (IQR, 3.8–37.4 months), and the median overall survival (mOS) was 10.1 months (95% CI: 7.8–12.1 months). Overall, 61.2% (*n* = 148/242) of these patients were considered frail, while 38.8% (*n* = 94/242) were nonfrail (Table 2). The percentage of frail patents was not significantly different between age groups (*p* = 0.12) or sex (female, *p* = 0.08; male, *p* = 0.14) (Table 3).

**Figure 2:**
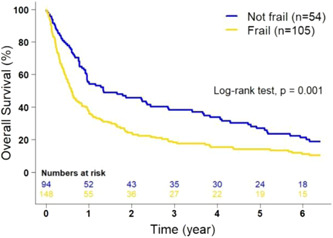
Kaplan–Meier estimates of overall survival of patients with pancreatic cancer according to frailty status as determined by the deficit accumulation frailty index (*p* = 0.001, adjusted).

Patients classified as frail had a significantly decreased mOS (7.1 months, 95% CI: 5.6–10.1) compared with nonfrail patients (16.1 months, 95% CI: 11.5–34.4) (*p* = 0.001), regardless of their treatment plan (Figure 2). Furthermore, multivariable Cox proportional‐hazards regression analyses demonstrated an association between frailty status and OS (aHR: 1.46; 95% CI: 1.11–1.92, *p* = 0.01), but no association was found between chronologic age and OS (aHR: 1.02; 95% CI: 0.99–1.04, *p* = 0.13) after adjusting for age, sex, race, and whether PC treatment included pancreatic resection (Table 4). Additionally, each 10% increase in DAFI was associated with a 21% increased risk of death (aHR: 1.21; 95% CI 1.10–1.34, *p* < 0.001).

### 3.4 Association of DAFI with OS in patients with PC according to surgery status

**Figure 3:**
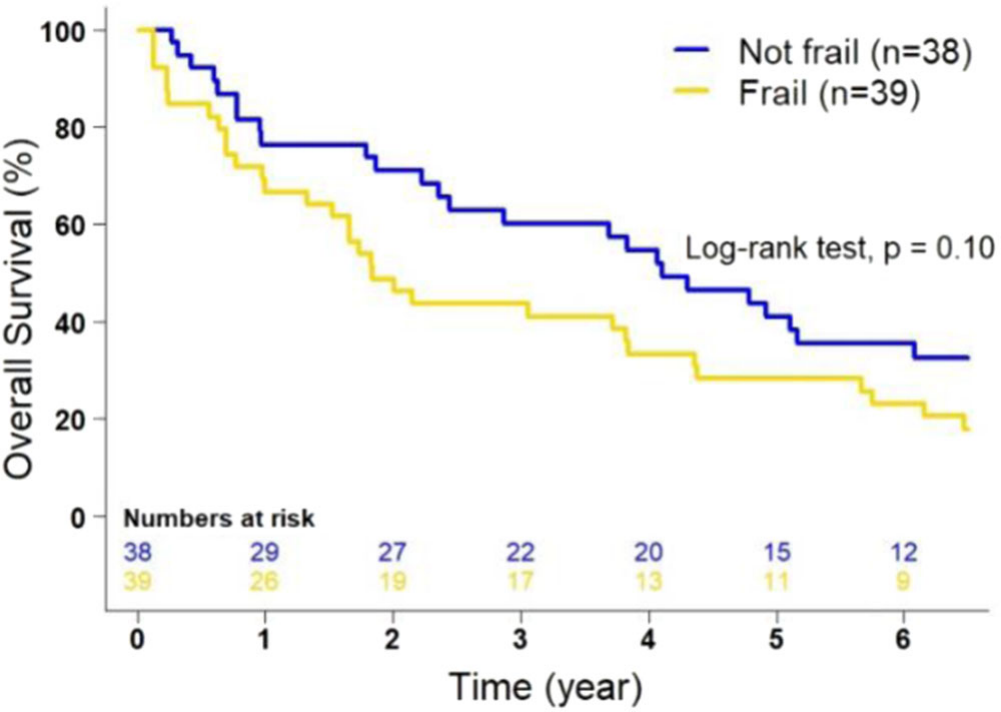
Kaplan–Meier estimates of overall survival of patients with pancreatic cancer who have undergone pancreatic resection (*n* = 77) according to frailty status (*p* = 0.100, adjusted). Frailty status was determined using the deficit accumulation frailty index.

**Figure 4:**
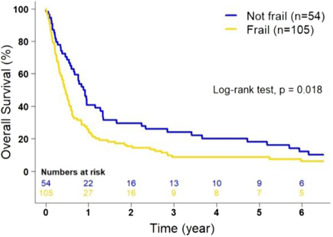
Kaplan–Meier estimates of overall survival of patients with pancreatic cancer who did not undergo pancreatic resection (*n* = 159) according to frailty status (*p* = 0.018, adjusted). Frailty status was determined using the deficit accumulation frailty index.

Subgroup analyses of the surgery and no surgery groups were performed to determine if the DAFI (frailty) was associated with OS. [Correction added on 18 January 2023, A sentence from this paragraph has been removed in this version]. Of the 236 patients with available surgical data, 32.6% (*n* = 77/236) of them underwent pancreatic resection (surgery group), while 67.4% (*n* = 159/236) of patients did not undergo pancreas resection (no surgery group) (Table 2). For the surgery group, patients classified as frail had a decreased mOS of 22.1 months (95% CI: 18.3–52.4) compared to that had by nonfrail patients (49.2 months, 95% CI: 29.3–79.9) with a trend toward statistical significance (*p* = 0.10; Figure 3); however, an association was identified after controlling for age, race, and sex (aHR: 1.45, 95% CI: 1.03–2.03, *p* = 0.03). For the nonsurgery group, those classified as frail also had a decreased mOS of 5.45 months (95% CI: 4.34–7.03) compared with that had by nonfrail patients (mOS 10.81 months, 95% CI: 7.85–16.03; *p* = 0.02; Figure 4).

## 4 DISCUSSION

Elderly patients with PC with a preserved biologic age (nonfrail) determined by our DAFI mathematical model should be treated with more aggressive treatment modalities. Our results demonstrate that nonfrail patients selected for pancreatic resection (surgery group) as part of their treatment plan had the most clinical benefit (mOS, 49 months), while frail patients had worse outcomes regardless of the treatment plan.

The difficulty in determining optimal treatment pathways for elderly patients with PC is that during PC treatment, the body endures stress resulting in limited physiologic reserve independent of chronologic age, and organ dysfunction may subsequently develop.^1^, ^2^, ^3^, ^4^, ^5^ Therefore, the biologic age of patients with PCs should be determined to better understand baseline physiologic reserves and tailor individual treatment recommendations appropriately rather than using the traditional measurements of frailty that are dependent on chronologic age.^6^, ^7^, ^8^, ^9^

Our study demonstrated the feasibility of constructing a DAFI tailored for elderly patients with pancreas cancer.^29^, ^30^ A strength of our study is that the methodology utilized adhered to the mathematical modeling used in previously validated non‐PC DAFI studies, which provide additional support to the development of a DAFI.^2^, ^23^, ^24^, ^25^ An interesting finding in our study was the slightly higher PC frailty rates (61.2% overall population) compared to nonpancreas cancer frailty rates (49% gynecologic cancers, 53% multiple myeloma, and 53.7% solid tumors), which may reflect the debilitating effect of PC on biologic age, patient tolerance of aggressive treatment regimens, and differences in biologic age among different cancers.^31^, ^32^, ^33^ Together, these findings suggest variability in the vulnerability of patients with cancer, supporting the need for cancer‐specific DAFIs to guide physicians in complex oncology management decisions.^39^

The mathematical algorithm used to create the DAFI for PC found an association with biologic age as a reflection of an individual's physiologic reserve independent of chronologic age. More importantly, the DAFI was associated with OS, but age was not. This finding may suggest that compared with other traditional static frailty indices (non‐DAFI) that are associated with chronologic age, DAFI is a better determinant of frailty in elderly patients with PC.^2^, ^9^, ^22^, ^26^, ^40^ This hypothesis is supported by previous DAFI studies that have demonstrated that biologic age is a more precise predictor of frailty than chronologic age. First, mathematical models that create a DAFI have improved our understanding of biologic age and provided more precise estimates of physiologic reserve by considering multiple factors outside of chronologic age and cancer, which influence the overall tolerability of treatment and long‐term outcomes.^2^, ^6^, ^23^, ^24^, ^25^, ^31^, ^32^, ^37^, ^38^, ^41^, ^42^, ^43^, ^44^, ^45^, ^46^, ^47^, ^48^ Therefore, estimating the biologic age for various cancers would enable us to better understand patients' baseline physiologic reserve and tailor treatment regimens appropriately.^2^, ^21^, ^23^, ^29^, ^39^, ^40^ Another major drawback of other frailty indices (non‐DAFI) is their reliance on pre‐existing medical conditions such as hypertension and chronic kidney disease, which are also static measures of frailty associated with chronologic age rather than biologic age.^9^, ^28^, ^30^ Finally, an additional weakness (non‐DAFI) is that they are static, do not change with time or changes in physiologic reserve (from diagnosis, during treatment, and recurrence), and lack medical interventions restricting their applicability to monitor treatment‐related frailty morbidity and tailor cancer treatment, a necessary feature of clinical frailty tools.

Our study evaluated pancreas resection outcomes (surgery group), a unique feature compared to previous cancer DAFI studies.^29^, ^31^, ^32^, ^33^ For the few patients who underwent surgical resection as part of their treatment plan (surgery group), those who showed a decline in biologic age tended to have a decrease in survival (mOS, 22 months) compared with that had by those who did not show a decline in biologic age (mOS, 49 months). Although this finding is not statistically significant, the clinical relevance of the predictive ability of the DAFI to determine treatment tolerability is substantial, with an improvement in survival of 27 months in nonfrail surgery patients (surgery group), suggesting its potential prognostic capability. This clinical improvement in survival is comparable to the recent PRODIGE 24‐ACCORD PC trial with an mOS of 54.5 months (FOLFIRINOX) and 35.0 months (Gemcitabine‐Abraxane), an unprecedented finding unlike that of any previous surgical randomized adjuvant PC trial (mOS, approximately 20–30 months).^4^, ^5^, ^49^ A criticism of the PRODIGE 24‐ACCORD trial was that the randomization was performed after pancreatic resections, which may bias the trial's results because the study included patients who could tolerate more aggressive treatments.^4^, ^5^, ^49^ However, this criticism supports our mathematical model's predictive ability to select elderly patients with PC who can tolerate more aggressive treatment plans comparable to the PRODIGE 24‐ACCORD inclusion criteria, which did not include elderly patients older than 79 years of age (median age, 65 years old) or frail patients (World Health Organization performance‐status score ≥2).^49^ Finally, the lack of statistical significance may be attributed to the small surgical population (*n* = 77), which was only 32.6% of the overall population and included frail (*n* = 39) and nonfrail (*n* = 38) cohorts. A larger study that is appropriately powered is needed to determine the association between DAFI and OS in patients who have undergone pancreatic resection.

To date, only a few predictive models have examined the association between frailty and pancreatic resection outcomes (surgery group), which we can use in comparison with our results.^9^, ^50^, ^51^, ^52^, ^53^, ^54^ Similar to our study, they have found an association between frailty and worse surgical outcomes.^9^, ^39^, ^40^, ^51^, ^55^, ^56^ Examples of indexes used in those studies include the American College of Surgeons National Surgical Quality Improvement Program (ACS‐NSQIP) modified frailty index (mFI‐11 and mFI‐5); the Canadian Study of Health and Aging‐Frailty Index (CSHA‐FI), and the Charlson Comorbidity Index (CCI),^50^, ^28^, ^51^, ^52^, ^53^, ^54^ which are older static indices of frailty (non‐DAFI) and have limitations that affect their ability to be used as a clinical tool as reported in the previous paragraph.^27^, ^28^, ^50^, ^51^, ^52^, ^53^, ^54^ More DAFI PC studies are needed and should include both systemic therapy and surgical outcomes to develop a DAFI predictive model to help physicians manage elderly patients with PC. Most importantly, it is necessary to identify which of the sequences of treatment (neoadjuvant vs adjuvant) is better suited for elderly patients with resectable PC (frail vs nonfrail) to improve their outcomes.^4^, ^5^, ^57^, ^58^

Our study is subject to the inherent limitations of retrospective studies (accuracy of the data and missing data points) and the nonprospective nature of the study design. A second limitation is the 10‐month median follow‐up, which may simply reflect the poor prognosis of patients with PC. Unfortunately, our study cannot determine the reasons for which treating physicians classify these patients as surgical or nonsurgical candidates, although there are several plausible factors (e.g., frailty, unresectable, metastatic disease, or patient and/or physician preference). Due to the constraints of available data within the SEER‐MHOS database, we could not assess the association of our PC DAFI with specific treatment regimens (e.g., treatment sequencing, specific chemotherapy regimens, and the dose of radiation), which may confound our findings. The SEER database, unfortunately, does not appropriately link this data to the MHOS data. Although we could not identify the reason behind physicians' decisions on the selection and sequencing of a given treatment plan, patients who had a decline in biologic age (frail patients determined by the DAFI) had a decreased mOS of 7.1 months compared with that had by to those who did not have a decline in biologic age (nonfrail: mOS, 16.1 months), regardless of their treatment plan.^5^ In addition, a 10% increase in the DAFI was associated with a 21% increase in the risk of death among the patients with PC. Furthermore, patient in the no surgery group had a worse biologic age (frail) and had a decrease in survival compared with that observed in those without a decline in biologic age (nonfrail) with a 5.45 mOS and 10.81 mOS, respectively. These results suggest our that the PC DAFI has prognostic ability regardless of the reason for selecting a given treatment plan. The final limitation of this study is the assumption that a MHOS survey completed within 1 year after a PC diagnosis accurately reflects baseline frailty at the time of a patient's diagnosis, as the initiation of treatment before survey completion may be a confounding factor. However, the authors believe it is a reasonable estimate to use in a feasibility study.

## 5 CONCLUSIONS

To our knowledge, this is the first SEER‐MHOS database study to examine the feasibility of constructing a DAFI in elderly patients with PC. Our mathematical model is unique in that it incorporated physiologic systems to determine biologic age (DAFI) and was less reliant on chronologic age, a weakness of previous static frailty indices. The need for computational frailty tools is further emphasized by the association of OS with frailty but not chronologic age. The validation of our PC DAFI is needed before clinicians can incorporate such findings into personalized complex oncology treatment plans to identify frail patients and initiate their pre‐habilitation program that will improve treatment tolerability and maximize the outcomes of elderly patients with PC.

## CONFLICT OF INTEREST

The authors declare no conflict of interest.

## SYNOPSIS

Biologic age as a marker of frailty was validated using mathematical modeling to determine the deficit accumulation frailty index (DAFI) in elderly patients with pancreatic cancer. The clinically relevant improvement in survival suggests that the DAFI may be used to select elderly patients for surgery.

## Data Availability

The data that supports the findings of this study are openly available in the SEER‐MHOS database at https://healthcaredelivery.cancer.gov/seer-mhos/.
